# Striatal D_1_ Dopamine Neuronal Population Dynamics in a Rat Model of Levodopa-Induced Dyskinesia

**DOI:** 10.3389/fnagi.2022.783893

**Published:** 2022-02-03

**Authors:** Shasha Gao, Rui Gao, Lu Yao, Jie Feng, Wanyuan Liu, Yingqiong Zhou, Qiongchi Zhang, Yong Wang, Jian Liu

**Affiliations:** ^1^Department of Physiology and Pathophysiology, School of Basic Medical Sciences, Institute of Neuroscience, Xi’an Jiaotong University Health Science Center, Xi’an, China; ^2^Department of Medical Imaging and Nuclear Medicine, The First Affiliated Hospital of Xi’an Jiaotong University, Xi’an, China

**Keywords:** levodopa, dyskinesia, D_1_ receptor, fiber photometry, optogenetics

## Abstract

**Background:**

The pathophysiology of levodopa-induced dyskinesia (LID) in Parkinson’s disease (PD) is not well understood. Experimental data from numerous investigations support the idea that aberrant activity of D_1_ dopamine receptor-positive medium spiny neurons in the striatal direct pathway is associated with LID. However, a direct link between the real-time activity of these striatal neurons and dyskinetic symptoms remains to be established.

**Methods:**

We examined the effect of acute levodopa treatment on striatal c-Fos expression in LID using D_1_-Cre PD rats with dyskinetic symptoms induced by chronic levodopa administration. We studied the real-time dynamics of striatal D_1_^+^ neurons during dyskinetic behavior using GCaMP_6_-based *in vivo* fiber photometry. We also examined the effects of striatal D_1_^+^ neuronal deactivation on dyskinesia in LID rats using optogenetics and chemogenetic methods.

**Results:**

Striatal D_1_^+^ neurons in LID rats showed increased expression of c-Fos, a widely used marker for neuronal activation, following levodopa injection. Fiber photometry revealed synchronized overactivity of striatal D_1_^+^ neurons during dyskinetic behavior in LID rats following levodopa administration. Consistent with these observations, optogenetic deactivation of striatal D_1_^+^ neurons was sufficient to inhibit most of the dyskinetic behaviors of LID animals. Moreover, chemogenetic inhibition of striatal D_1_^+^ neurons delayed the onset of dyskinetic behavior after levodopa administration.

**Conclusion:**

Our data demonstrated that aberrant activity of striatal D_1_^+^ neuronal population was causally linked with real-time dyskinetic symptoms in LID rats.

## Introduction

Parkinson’s disease (PD) is a progressive neurodegenerative disorder that causes progressive motor deficits. The principal pathological characteristic of PD is the progressive death of dopaminergic neurons in the substantia nigra pars compacta (SNc) (Ehringer and Hornykiewicz, 1960; Graybiel et al., 1990). Levodopa, the dopamine precursor molecule, is the mainstay of symptomatic treatment for PD (Pezzoli and Zini, 2010). Unfortunately, the positive effects of levodopa in PD often lead to the development of adverse motor fluctuations. Levodopa-induced dyskinesia (LID) is the most debilitating motor fluctuation after chronic administration of levodopa (Nadjar et al., 2009; Calabresi et al., 2010). A better insight into the pathophysiology of LID is valuable for improvements in its prevention and treatment.

The striatum is one of the main subcortical components that controls voluntary movement (Nakano et al., 2000). Neuronal activity in the striatum is affected by glutamatergic fibers from the cerebral cortex and dopaminergic afferents from the SNc (Blandini et al., 2000). The medium spiny projection neurons (MSNs) of the striatum can be divided into two populations based on the type of dopaminergic receptor expression. Approximately half of these neurons express the dopaminergic D_1_ receptor and project monosynaptically to the basal ganglia output nuclei, forming the direct pathway. The remaining half of these neurons are D_2_ receptor-expressing neurons that form the polysynaptic indirect pathway (Alexander and Crutcher, 1990; Blandini et al., 2000). Activation of D_1_^+^ neurons in the direct pathway is believed to facilitate movement and their function is affected by many pathological and pharmacological factors (Mink, 2003; Cui et al., 2013). Previous studies have indicated that PD and LID are associated with aberrant activity in the direct and indirect striatal pathways (Blandini et al., 2000; Picconi et al., 2003; Calabresi et al., 2010; F Hernández et al., 2017; Parker et al., 2018). Experimental data from numerous investigations support the idea that increased striatal D_1_ dopamine receptor signaling is crucially involved in the molecular pathology underlying LID (Aubert et al., 2005; Pavón et al., 2006; Santini et al., 2008; Darmopil et al., 2009; Calabresi et al., 2010). Dopamine depletion in PD reduces the firing rate of D_1_^+^ striatal MSNs. In contrast, levodopa increases the firing rates of D_1_^+^ striatal MSNs and optogenetic activation of these neurons can induce dyskinesia-like symptoms in a mouse model of PD (Perez et al., 2017; Parker et al., 2018; Ryan et al., 2018; Keifman et al., 2019). However, real-time relationship between the activity of striatal D_1_^+^ MSNs and specific dyskinetic symptoms in LID animals remains unclear.

To address this issue, we used GCaMP-based fiber photometry to optically record the real-time activity of striatal D_1_^+^ neuronal populations following levodopa administration in freely behaving LID rats. Subsequently, we silenced these neurons using optogenetic and chemogenetic methods to assess the effects of striatal D_1_^+^ neuronal deactivation on levodopa-induced dyskinetic symptoms in LID rats. We found that the activity of striatal D_1_^+^ neuronal population was causally linked with dyskinetic symptoms. Our results suggest a behavior-relevant cell-type-specific neuronal mechanism of LID.

## Materials and Methods

### Animals

Heterozygous Drd1-Cre transgenic rats (Sprague-Dawley genetic background) from Biocytogen (Beijing Biocytogen Co., Ltd., Beijing, China) were used in this study (Yu et al., 2019). To ensure that the rats had a consistent genetic background, we crossed the Drd1-Cre rats with wild-type rats for at least six generations. The genotype of the transgenic Drd1-Cre rat offspring was identified by polymerase chain reaction test on genomic tail DNA. Male rats 8–10 weeks of age and weighing 230–250 g were enrolled in the study. They were group-housed on a 12-h light/dark cycle with *ad libitum* access to water and rodent chow. All experimental protocols complied with the ARRIVE guidelines and were strictly conducted according to the National Institutes of Health Guide for the Care and Use of Laboratory Animals (NIH Publications No. 8,023, revised 1978) and were approved by the Ethics Committee for Animal Experimentation of Xi’an Jiaotong University. All efforts were made to minimize the number of animals used and to reduce their suffering.

### Stereotaxic Animal Surgery and Levodopa Administration

**FIGURE 1:**
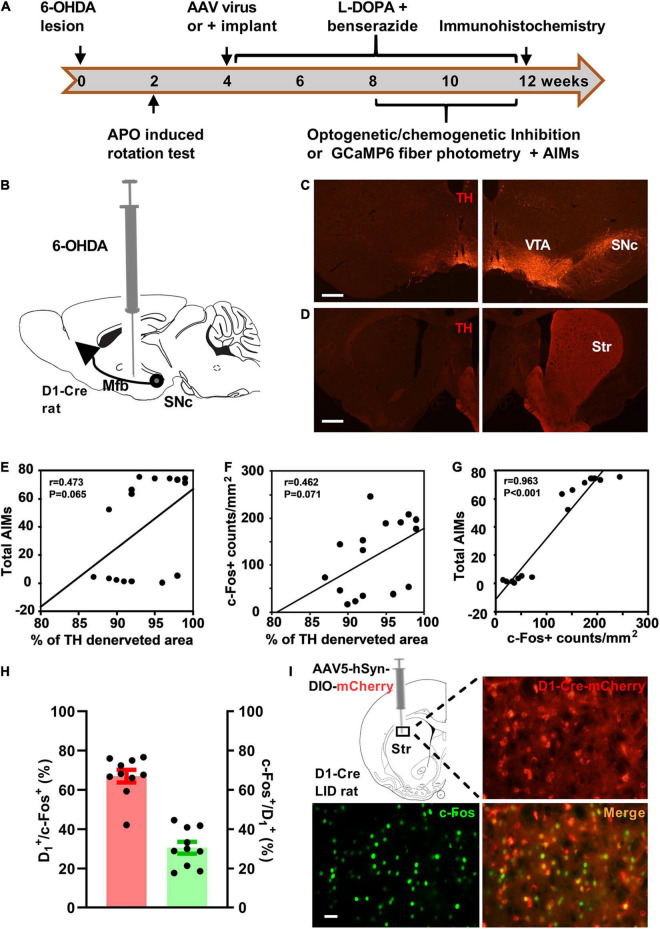
Striatal D_1_ receptor-positive neurons were activated in the rat model of levodopa-induced dyskinesia (LID). **(A)** Experimental design of the study. **(B)** Dopaminergic lesion was induced by stereotaxic injection of 6-hydroxydopamine (6-OHDA) in the left medial forebrain bundle (Mfb). Photomicrographs of tyrosine hydroxylase (TH) staining of substantia nigra pars compacta (SNc, **C**) and striatum (Str, **D**) on the injected side (left; scale bar = 500 μm) and contralateral intact side (right). Simple linear regression analysis illustrating the correlation between the percentage of total striatum dopaminergic denervation and the total Abnormal Involuntary Movement scale (AIMs) scores (**E**; *n* = 16) or striatal c-Fos^+^ neuron counts/mm^2^ (**F**; *n* = 16). Linear regression analysis illustrating the correlation between striatal c-Fos^+^ neuron counts/mm^2^ and the AIMs scores (**G**; *n* = 16). Quantification (**H**; red: percentage of c-Fos^+^ neurons positive for D_1_-Cre-mCherry, green: percentage of D_1_-Cre-mCherry^+^ neurons positive for c-Fos) and representative histology (**I**, scale bar = 50 μm) of c-Fos immunoreactivity in the striatal brain section of D_1_-Cre-mCherry LID rats after levodopa administration. APO, apomorphine; Cre, cyclization recombinase; AAV, adeno-associated virus; AIMs, Abnormal involuntary movement scale; VTA, ventral tegmental area.

Rats were treated as shown in Figures 1A,B. All surgeries were performed with a stereotaxic frame using aseptic techniques. Rats were anesthetized with 5% isoflurane and anesthesia was maintained with 1–2% isoflurane. Unilateral injection of 6-hydroxydopamine hydrochloride (6-OHDA; dissolved in ice-cold normal saline containing 0.02% ascorbic acid, 12 μg/4 μl) was administered to the rats in the left medial forebrain bundle (coordinates: Anterior-posterior, -4.20 mm; Lateral, -1.25 mm; Dorsal, -7.80 mm relative to the bregma and the dural surface; (Paxinos and Watson, 2006) as previously described (Lindgren et al., 2010; Wang et al., 2014). Effects of the dopaminergic lesion were verified using apomorphine-induced (0.05 mg/kg, s.c.) contralateral circling test 2 weeks after the surgery (Wang et al., 2014).

**FIGURE 2:**
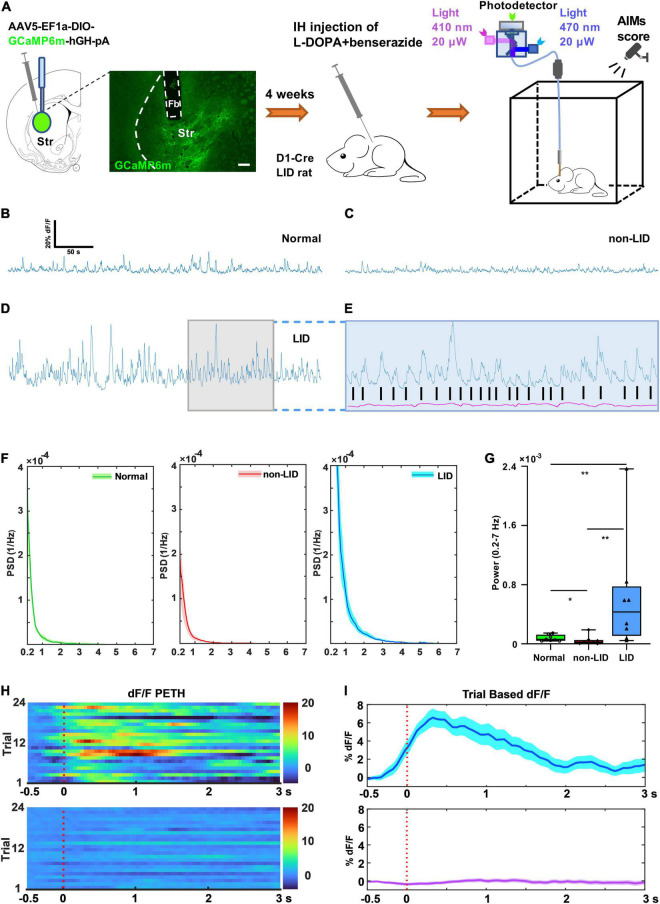
Striatal D_1_^+^ neuronal population activity during dyskinetic period in rats with levodopa-induced dyskinesia (LID). **(A)** Experimental setup for the GCaMP_6m_-based fiber photometry assay (scale bar = 200 μm). Representative striatal D_1_^+^ GCaMP signal (dF/F, 300 s) of a normal control **(B)**, non-LID rat **(C)**, and LID rat **(D)** obtained at 30–80 min after levodopa injection. **(E)** Photometry traces from isosbestic control (410 nm, purple) and GCaMP (blue) of a LID rat showing robust increases in GCaMP fluorescence (dF/F), which correlated with the onsets of each dyskinetic movement (black vertical lines). **(F)** Power spectral density (PSD) plots of GCaMP fluorescence traces (300 s, dF/F) obtained from normal, non-LID, and LID rats (data are presented as mean ± standard error of the mean, *n* = 8 for each group). **(G)** Power (at 0.2–7 Hz over 5 min) obtained from GCaMP fluorescence traces (dF/F) of normal, non-LID and LID rats (*n* = 8 for each group, Kruskal–Wallis one way analysis of variance on ranks with *post-hoc* Student–Newman–Keuls Method: *H*_2_ = 12.560, *P* = 0.002; LID vs. non-LID: *q* = 5.000; LID vs. normal: *q* = 4.159; Normal vs. non-LID: *q* = 3.267; *P < 0.05, **P < 0.01). Representative heatmaps **(H)** and peri-event time histograms (PETH, **I**) aligned to the start of each dyskinetic movement for GCaMP (top) or isosbestic control (bottom) traces. SC, subcutaneous.

**FIGURE 3:**
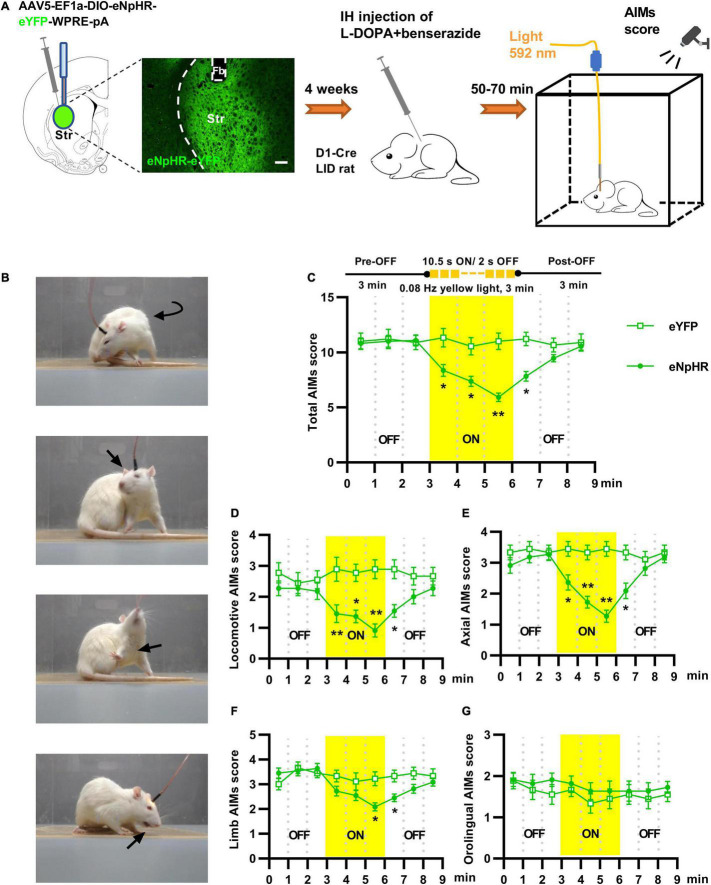
Optogenetic inhibition of striatal D_1_^+^ neurons suppressed abnormal involuntary movements of rats with levodopa-induced dyskinesia (LID). **(A)** Experimental design of the optogenetic assay using stereotaxic injection of Cre-dependent AAV to express eNpHR_3.0_ or eYFP in striatal D_1_^+^ neurons (scale bar = 200 μm). **(B)** Photographs from LID rats affected by locomotive, axial, forelimb and orolingual abnormal involuntary movements. **(C)** Change in the total Abnormal Involuntary Movement scale (AIMs) scores before, during, and after striatal yellow light (592 nm) in eNpHR3.0 or eYFP rats [*n* = 11 for eNpHR group and *n* = 9 for eYFP control; two-way repeated measures analysis of variance (ANOVA) with *post-hoc* Holm-Sidak method: *F*_(1, 18)_ = 5.910, *P* = 0.026; *P < 0.05, **P < 0.01]. Effects of striatal yellow light on locomotive **(D)**, axial **(E)**, forelimb **(F)** and orolingual **(G)** AIMs score (two-way repeated measures ANOVA with *post-hoc* Holm-Sidak method; **(D)** *F*_(1, 18)_ = 10.389, *P* = 0.*005*; **(E)** *F*_(1, 18)_ = 9.821, *P* = 0.006; **(F)** *F*_(1, 18)_ = 3.649, *P* = 0.072; **(G)** *F*_(1, 18)_ = 1.219, *P* = 0.284; *P < 0.05, **P < 0.01.

The vector virus was injected into the dorsal striatum (Anterior-posterior, + 0.6 mm; Lateral, -3.6 mm; Dorsal, -3.6 mm; (Paxinos and Watson, 2006) on the side ipsilateral to the 6-OHDA lesion at 1-2 weeks after the apomorphine test. AAV5-EF1a-DIO-EYFP-WPRE-pA, AAV5-EF1a-DIO-eNpHR3.0-EYFP-WPRE-pA, AAV5-EF1a-DIO-GCaMP6m-WPRE-hGH-pA, AAV5-hSyn-DIO-mcherry and AAV5-hSyn-DIO-hM4Di-mCherry were deposited and packaged into viral vectors (BrainVTA Co., Ltd.; Hubei, China). The injection volume was 300-nl. For optogenetics and fiber photometry tests, optical ferrule fibers (outer diameter: 220 μm, OD, numerical aperture [NA]: 0.37) were implanted 0.3-0.5mm above the injection coordinates immediately after virus infusion (Figures 2A, 3A). After the implantation, dental cement (Super-Bond C&B, Sun Medical, Shiga, Japan) was used to secure the fiber to the skull. At least 4 weeks after the surgery, recovery of the animals and viral expression were allowed before the behavioral assays (Wang et al., 2019). One week after virus injection, levodopa plus benserazide (L-3, 4-dihydroxyphenylalanine methyl ester hydrochloride, 6 mg/kg; benserazide hydrochloride, 12 mg/kg, s.c; Sigma-Aldrich, St. Louis, United States) were injected once daily for 21 days (Lindgren et al., 2010). After 3 weeks of chronic levodopa treatment, each rat was observed every 20 min for 180 min and scores were assigned using the standard procedures of Abnormal Involuntary Movement scale (AIMs) for four subtypes of dyskinesia including axial, limb, orolingual, and contralateral circular locomotion on a scale from 0 to 4 (Figure 3B). The maximum AIMs score per time-point was 16 (Winkler et al., 2002). Rats with apparent dyskinetic symptoms and high AIMs scores were classified into the LID group, and rats with no apparent abnormal involuntary movements were classified into the non-dyskinetic (non-LID) group (Lindgren et al., 2010).

**FIGURE 4:**
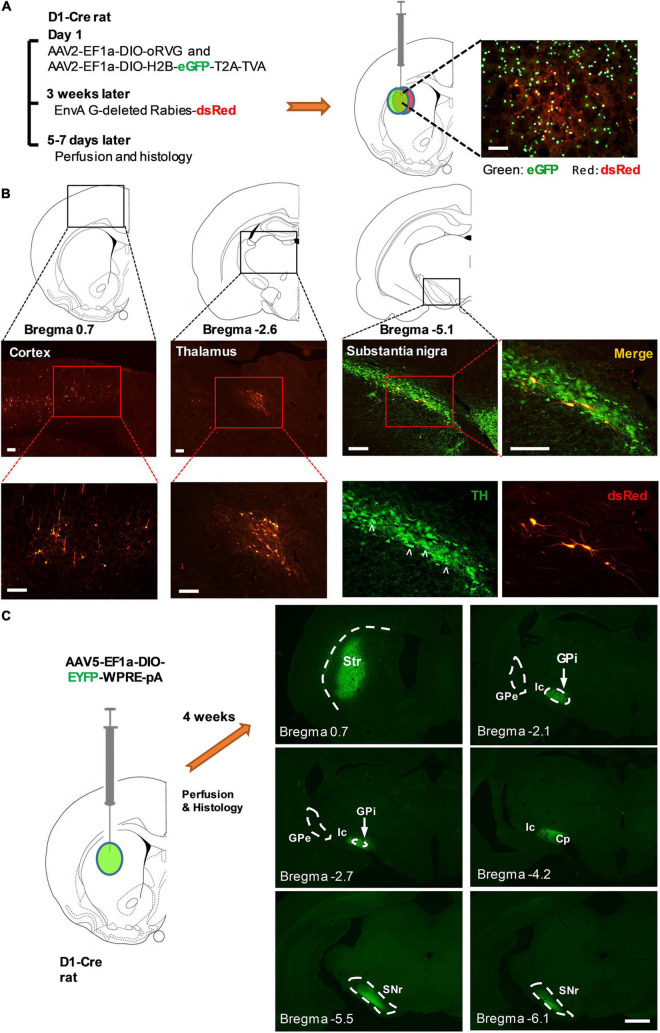
Striatal D_1_-Cre^+^ neurons received monosynaptic inputs from multiple brain regions and sent fibers directly to the major output nuclei of the basal ganglia. **(A)** Diagram illustrating the procedure of virus injection for monosynaptic retrograde rabies virus tracing (left). Striatal D_1_-Cre^+^ neurons expressing eGFP and dsRed as starter cells for retrograde tracing (right). **(B)** Major brain regions that send monosynaptic innervation to Striatal D_1_-Cre^+^ neurons including the cortex (left), thalamus (middle), and the substantia nigra pars compacta (right). TH: tyrosine hydroxylase, scale bar = 200 μm. **(C)** Fluorescent nerve terminals from striatal D_1_^+^ neurons. Striatal D_1_^+^ neurons were labeled using the injection of AAV5-EF1a-DIO-EYFP-WPRE-pA in the striatum of D_1_-Cre rats. Str, striatum, GPi, internal globus pallidus, GPe, external globus pallidus, Ic, internal capsule, Cp, cerebral peduncle, SNr, substantia nigra pars reticulata (scale bar = 1,000 μm).

For monosynaptic rabies virus tracing, AAV2-EF1a-DIO-oRVG-WPRE-hGH-pA and AAV2-EF1a-DIO-H2B-eGFP-T2A-TVA-WPRE-hGH-pA (BrainVTA) were mixed at a ratio of 1:3 and then injected in the striatum. About 3 weeks later, EnvA G-deleted Rabies-dsRed (BrainVTA) was injected in the same place (Figure 4A; Callaway and Luo, 2015).

### GCaMP6-Based Calcium Fiber Photometry

A dual-color multichannel fiber photometry system (Inper Hangzhou Bioscience Inc., Hangzhou, China) was used to detect and record the fluorescence signals of GCaMP6m. The recording mode is shown in Figure 2A. An optical fiber was integrated with a fiber-optic rotary joint for fiber photometry (FRJ_1 × 1_PT, Doric Lenses, Quebec, Canada) to guide the light between the fiber photometry system and the implanted optical ferrule fiber. Lights of 470 and 410 nm wavelengths from a light-emitting diode (Thorlabs Inc., Newton, NJ, United States) were bandpass filtered and delivered to the brain as excitation sources for the Ca^2+^-dependent and Ca^2+^-independent control measurements, respectively. Light intensity at the top of the fiber was set to 20 μW to minimize bleaching. Time-division multiplexing was implemented to acquire Ca^2+^-dependent GCaMP signals and Ca^2+^-independent control signals simultaneously (Gunaydin et al., 2014; Kim et al., 2016). The emission light that traveled through the same optical fiber was bandpass filtered (passing band: 535 ± 25 nm) and captured by a scientific complementary metal-oxide semiconductor camera (Basler; Ahrensburg, Germany) with a sampling frequency of 40frames/s (Figure 2A). Streamline fiber photometry data and real-time rat behavioral videos from a high-speed digital video camera (Logitech, Shanghai, China) were synchronized with Inper Studio data acquisition (Inper Hangzhou Bioscience Inc., Hangzhou, China). The initiation time for each dyskinetic event was determined using video scoring. The raw data of fiber photometry and real-time behavioral videos were saved in CVS and MP4 files, respectively.

Analysis was performed using custom MATLAB (MathWorks, Portola Valley, CA, United States) scripts and Photometry Modular Analysis Tool (pMAT) (Bruno et al., 2021). We subtracted the scaled 410-nm reference trace from the 470-nm signal to obtain the motion-corrected 470-nm signal. We calculated the normalized change in motion-corrected 470-nm signal (dF/F) by subtracting the median signal from the signal at each time point and dividing that value by the median signal. To calculate peri-event time heatmaps and histograms in the pMAT suite, the event window was set around the initiation time for each dyskinetic event in LID rats (Li et al., 2016; Vesuna et al., 2020). The baseline window was defined as 0.5 s preceding each event window (Figures 2F,G). The corresponding power spectral density (PSD) of the GCaMP signal (dF/F, 300 s) was then estimated using Welch’s method [pwelch() in MATLAB], with a window size of 10 fs (sampling rate of the signal). The average band power within the dominant frequency band (0.2–7 Hz) was then computed by integrating the PSD estimate [bandpower() in MATLAB] (Vesuna et al., 2020).

### *In vivo* Optogenetics and Chemogenetics

A yellow laser (Aurora120–589, 589 nm, 50 mW; Newdoon Inc., Hangzhou, China) was used to deliver light for optogenetic inhibition. A stimulus generator (STG4002, Multi-Channel Systems, Reutlingen, Germany) was used to control the frequency and pulse width of the laser light. The light was delivered to the brain through an optical fiber (200 μm diameter, NA: 0.37) connected to the implanted ferrule fiber using a zirconium sleeve. The light power in the brain regions 0.5 mm below the fiber tip was calibrated as described previously (Wang et al., 2019). The calibrated light power density (0.5 mm below the fiber tip) used in light deactivation experiment was 5 mW/mm^2^ (Gradinaru et al., 2008; Stefanik et al., 2013; Lee et al., 2017). At 50–70 min after the administration of levodopa, the LID rats were placed individually in the center of the box and their dyskinetic behavior was tracked for 9min in optogenetic tests with 3 min of light inhibition (589 nm, 10.5 s-ON/2 s-OFF pulse, 0.08 Hz) applied at 3 min after the start (Figure 3A). All behaviors were videotaped and analyzed offline.

**FIGURE 5:**
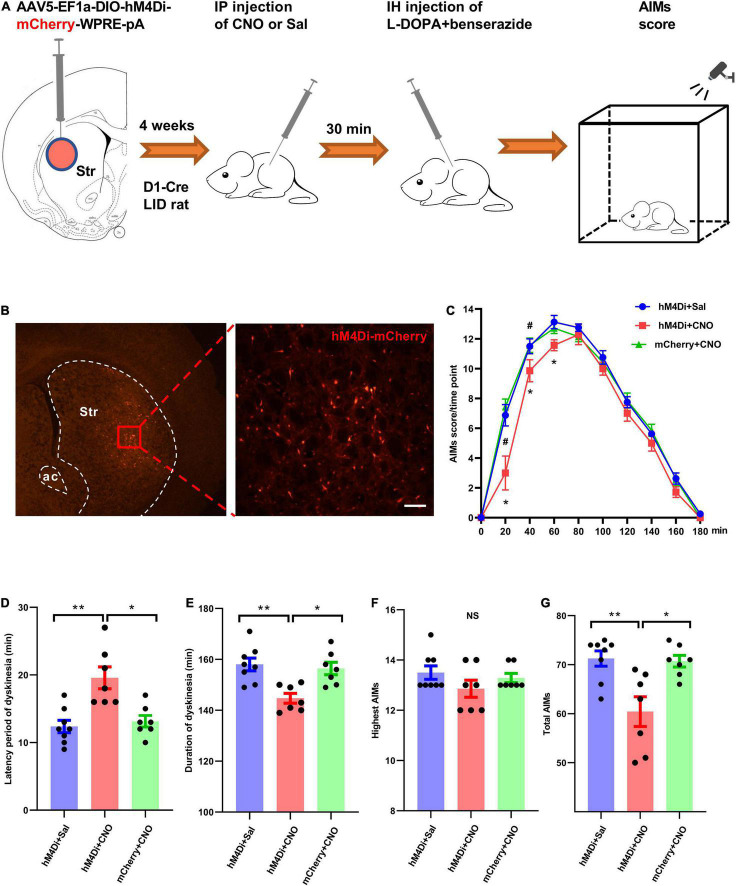
Chemogenetic silencing of striatal D_1_^+^ neurons suppressed dyskinetic movements in rats with levodopa-induced dyskinesia (LID). **(A)** Experimental design of the chemogenetic assay using stereotaxic injection of Cre-dependent AAV to express hM_4_Di-mCherry or mCherry in striatal D_1_^+^ neurons. **(B)** Representative photomicrographs of striatal brain slice of a D_1_-Cre rat following intra-striatal infusion of AAV-DIO-hM4Di-mCherry. **(C)** The time-course plot of Abnormal Involuntary Movement scale (AIMs) scores per time point at intervals of 20 min following levodopa administration and intraperitoneal injection of clozapine-N-oxide (CNO) or saline (Sal) in dyskinetic rats from different groups (*n* = 8 for hM_4_Di+Sal group, *n* = 7 for hM_4_Di+CNO group and mCherry+CNO group; two-way repeated measures analysis of variance (ANOVA) with *post-hoc* Holm-Sidak method: *F*_(2, 219)_ = 8.641, *P* = 0.002; hM_4_Di+CNO vs. hM_4_Di+Sal: *P < 0.01; hM_4_Di+CNO vs. mCherry+CNO: ^#^*P* < 0.01). Comparison of different parameters of dyskinetic behaviors among hM_4_Di+Sal (*n* = 8), hM_4_Di+CNO (*n* = 7), and mCherry+CNO (*n* = 7) groups (**D–G**; One-way ANOVA with *post-hoc* Holm-Sidak method: *F*_(2, 21)_ = 11.361, *P* < 0.001 **(D)**; *F*_(2, 21)_ = 9.402, *P* = 0.001 **(E)**; *F*_(2, 21)_ = 1.457, *P* = 0.258 **(F)**; *F*_(2, 21)_ = 8.744, *P* = 0.002 **(G)**; *P < 0.01, **P < 0.001, NS, not significant; IP, intraperitoneal.

Clozapine-N-oxide (CNO, BML-NS105-0005; Enzo Life Sciences Inc., Farmingdale, United States) was freshly dissolved in saline (0.9% NaCl; 1 mg/ml). It was injected intraperitoneally at 5 mg/kg for hM4Di silencing. Behavioral tests were usually performed 30-40 min after CNO injection and immediately after levodopa administration. Saline was injected as the vehicle control. Dyskinetic behavior was tracked for 180 min in the chemogenetic tests (Figure 5A).

### Histology and Immunohistochemistry

The rats were administered an overdose of 20% urethane at 80-100 min after the last levodopa administration and transcardially perfused with 200 ml of 0.01 M phosphate-buffered saline (PBS), followed by 300-ml of 4% paraformaldehyde. Brains were removed and placed in 4% paraformaldehyde for 8 h followed by immersion in 30% sucrose solution until sinking was observed. Brains were sectioned in 30 μm in the coronal plane using a cryostat slicer. The sections mounted on glass slides were used to identify the anatomical placement of the optical fibers and virus expression. To determine the extent of dopaminergic neuron degeneration in following injection of 6-OHDA, tyrosine hydroxylase (TH) immunofluorescence histochemistry of the SNc and striatum was performed (Figures 1C,D). Only the rats with almost total loss (>97%) of TH immunoreactivity in the left SNc were used to analyze the data. In addition, immunofluorescence histochemistry was used to observe the localization of c-Fos or D_1_ staining in the striatum. The sections were incubated for 24 h at 4°C in monoclonal mouse anti-c-Fos primary antibody-containing solution (1:1,200; ab208942, Abcam, Cambridge, United Kingdom), rabbit polyclonal antibody for dopamine D_1_ receptor (1:500; ab40653, Abcam) or polyclonal rabbit anti-TH primary antibody-containing solution (1:1,200; ab112, Abcam). After several washes, the sections were incubated with donkey anti-mouse IgG H&L (Alexa Fluor 488, 1:500; ab 150073, Abcam) or donkey anti-rabbit IgG H&L (Alexa Fluor 594, 1:1,000; ab 150108, Abcam) antibodies for 2 h at room temperature. The sections were then washed in PBS, mounted, and coverslipped using Fluoromount-G gel (SouthernBiotech, Birmingham, United Kingdom). The digital images were captured from the sections using a fluorescent microscope (BX51, Olympus, Tokyo, Japan). Co-localization and quantification of the fluorescence images were performed using the cell counter plug-in in ImageJ.

### Quantification and Statistical Analysis

Data were represented as mean ± standard error of the mean or median as indicated. One-way analysis of variance (ANOVA) with *post-hoc* Holm-Sidak method or Kruskal–Wallis One-way ANOVA on ranks with *post-hoc* Student–Newman–Keuls method was used to compare three or more groups with a single variable. Two-way repeated measures ANOVA with *post-hoc* Holm-Sidak method was used for data with more than one independent variable over more than two time points. Statistical significance was set at *P* < 0.05. Data were analyzed using GraphPad Prism 8.0 (GraphPad Software, San Diego, United States) and SigmaStat 3.5 (Systat Software, Inc., San Jose, United States).

## Results

### The Severity of Levodopa-Induced Dyskinesia Is Correlated With Striatal c-Fos Expression After Levodopa Administration

Following unilateral 6-OHDA lesion of the MFB and chronic levodopa (6 mg/kg) treatment (Figure 1A), we evaluated the effect of striatal dopaminergic denervation on total AIMs scores and striatal c-Fos expression at 80–100 min after subcutaneous injection of levodopa. The administration of levodopa (Figure 1I), but not saline (Supplementary Figure 1), increased striatal c-Fos expression of LID rats. The c-Fos expression after levodopa injection was confined in unilateral striatal areas with complete dopaminergic denervation. However, the percentage of striatal area with complete dopaminergic lesion showed no significant correlation with the total AIMs scores and striatal c-Fos^+^ neurons counts per mm^2^ (Figures 1E,F) after almost complete TH denervation on ipsilateral striatum (Figure 1D). In contrast, the striatal c-Fos^+^ neurons counts/mm^2^ strongly correlated with the total AIMs scores (Figure 1G).

### Striatal D_1_ Receptor-Positive Neurons of Dyskinetic Rats Were Activated Following Levodopa Administration

To investigate the involvement of striatal D_1_ receptor-positive neurons in the regulation of LID, we monitored c-Fos expression in D_1_^+^ neurons after levodopa administration. The D_1_-Cre LID rats were used in the assay at 4 weeks after intra-striatal injection of AAV5-hSyn-DIO-mCherry. Immunostaining for c-Fos revealed that levodopa administration induced substantial c-Fos expression in the striatal neurons of dyskinetic rats and D_1_-Cre-mCherry marked a sizeable fraction of c-Fos^+^ neurons activated by levodopa (Figure 1I). Notably, the majority (66.97%) of the c-Fos^+^ striatal neurons activated by levodopa were positive for D_1_-Cre-mCherry and 30.58% of the D_1_-Cre-mCherry ^+^ striatal neurons showed c-Fos expression (Figure 1H).

In addition, we used the Cre-dependent monosynaptic retrograde rabies system to screen for the brain regions that send inputs to striatal D_1_-Cre^+^ neurons (Figure 4A). Projection neurons from the cortex and thalamus, and dopaminergic neurons from the SNc were identified as the monosynaptic upstream of D_1_-Cre^+^ neurons in the striatum (Figure 4B). An anterograde tracing study showed that striatal D_1_-Cre-eYFP^+^ neurons sent their fibers directly to the basal ganglia output nuclei, internal globus pallidus, and substantia nigra pars reticulata. In contrast, D_1_-Cre-eYFP^+^ fibers were sparse in the external globus pallidus (Figure 4C). The results confirmed that striatal D_1_-Cre^+^ neurons were involved in the direct pathway of the basal ganglia.

### Striatal D_1_^+^ Neuronal Population Activity During Dyskinetic Behavior in Levodopa-Induced Dyskinesia Rats

We investigated how striatal D_1_^+^ neurons responded during the dyskinetic phase in LID rats. *In vivo* calcium imaging with fiber photometry was used to record striatal D_1_^+^ neuronal population activity of D_1_-Cre LID and non-LID rats following levodopa injection and D_1_-Cre normal rats without 6-OHDA lesion and levodopa administration. We virally expressed the genetically encoded calcium indicator GCaMP_6m_ in the D_1_^+^ neurons of the striatum ipsilateral to the 6-OHDA lesion. GCaMP_6m_ expression was largely limited to the dorsolateral striatum (Figure 2A). We recorded stable striatal D_1_^+^ neuronal GCaMP fluorescence signal from normal controls, non-LID rats, and LID rats following levodopa administration (Figures 2B–D). The signal of striatal D_1_^+^ neuronal population activity from all three groups was mainly distributed in the low frequency band (0.2–7 Hz; Figure 2F). In contrast, the magnitude of striatal D_1_^+^ neuronal GCaMP fluorescence signal was different among the three groups (Figures 2B–D).

Quantitative analysis showed that the average power (0.2–7 Hz) of striatal D_1_^+^ neuronal GCaMP fluorescence signal in LID rats was significantly higher than that in non-LID and normal controls (Figure 2G). These findings indicated that the striatal D_1_^+^ neurons in LID rats were overactivated by levodopa administration. However, it was unclear whether the neurons would be activated during each dyskinetic phase. To address this issue, we examined the temporal response profiles during the dyskinetic phases in LID rats by plotting heatmaps and peri-event time histograms (PETHs) of D_1_^+^ neuronal GCaMP fluorescence signals aligned with the initiation of each dyskinetic movement. Sorting the population activity of striatal D_1_^+^ neurons by the initiation time of dyskinetic movements revealed that the striatal D_1_^+^ neuronal population activity peaked at each dyskinetic movement in LID rats (Figures 2E,H,I and Supplementary Video 1). We observed no associated changes in Ca^2+^-independent control fluorescence signals during dyskinetic behavior, indicating that the contribution of movement-induced artifacts to the fluorescence signal was negligible (Figures 2H,I; Kim et al., 2016).

### Optogenetic Deactivation of Striatal D_1_^+^ Neurons Reduced Dyskinetic Behavior in Levodopa-Induced Dyskinesia Rats

Overactivity of striatal D_1_^+^ neuronal population was observed during dyskinetic movements in LID rats, but the functional role of striatal D_1_^+^ neurons in levodopa-induced dyskinetic behavior has not been fully explored. To address this issue, we virally expressed eNpHR3.0, a fast light-activated electrogenic Cl^–^ pump, unilaterally in the striatal D_1_^+^ neurons of LID rats (Figure 3A). To determine the effects of striatal D_1_^+^ neuronal deactivation on dyskinetic movements in LID rats, we delivered yellow light when the animals showed stable and severe dyskinetic symptoms (Figures 3A,B). When compared with the eYFP controls, optogenetic inhibition of striatal D_1_^+^ neurons reduced locomotive, axial, limb, and total AIMs scores of eNpHR3.0-expressing LID rats (Figures 3C–F and Supplementary Video 2). However, yellow light did not affect the orolingual AIMs scores of eNpHR3.0–expressing LID rats (Figure 3G). These results suggest that the overactivity of striatal D_1_^+^ neurons is necessary for most of the dyskinetic symptoms in LID rats.

### Chemogenetic Inhibition of Striatal D_1_^+^ Neurons Delayed the Onset of Dyskinetic Behavior After Levodopa Administration

We tested whether long-term inhibition of striatal D_1_^+^ neuronal population activity in LID rats could affect their dyskinetic behavior after levodopa injection. A G_*i*_-coupled designer receptor exclusively activated by designer drugs (DREADDi) conjugated with mCherry was expressed unilaterally in striatal D_1_^+^ neurons following intra-striatal microinfusion of AAV5-Syn-DIO-hM4Di-mCherry-WPRE-pA (Figures 5A,B). AAV5-Syn-DIO-mCherry-WPRE-pA was used as a control. Following selective binding to CNO, DREADDi could hyperpolarize hM4Di-mCherry-expressing neurons (Dong et al., 2010). We examined the effect of striatal D_1_^+^ neuronal deactivation on dyskinetic behaviors of LID rats by alternating injections of saline and CNO (5 mg/kg of body weight). LID rats were observed for 180 min and AIMs scores were assigned after systemic injections of CNO/saline and levodopa (Figures 5A,C). A two-way repeated measures ANOVA revealed significant overall differences among the three groups [*F*_(2, 219)_ = 8.641, *P* = 0.002] and across time points [*F*_(9, 219)_ = 381.419, *P* < 0.001], as well as a significant interaction between these parameters [*F*_(18, 219)_ = 2.767, *P* < 0.001]. In *post-hoc* comparisons (Holm-Sidak method), AIMs scores in the hM4Di+CNO group were significantly lower than those in the hM4Di+Sal and mCherry+CNO groups (*P* = 0.002 and *P* = 0.003, respectively; Figure 5C). In addition, we found that CNO injection significantly reduced the dyskinetic behavior of hM4Di-expressing LID rats in the early phase. Administration of CNO significantly increased the latency period of dyskinesia expression in hM4Di+CNO LID rats after levodopa injection when compared with hM4Di+Sal and mCherry+CNO groups (Figures 5C,D). CNO-mediated deactivation significantly reduced the duration of dyskinesia and the total AIMs score in hM_4_D_*i*_-expressing LID rats, but did not affect the highest AIMs score (Figures 5E–G).

## Discussion

In the present study, we applied anatomical and functional methodologies to probe the striatal neuronal correlates of LID in rats. Through a series of experiments, we identified the abnormal dynamics of the striatal D_1_^+^ neuronal population as a potential neural mechanism underlying LID.

It is well accepted that aberrant activation of the striatum is involved in the pathophysiology of LID. Administration of levodopa induces expression c-Fos and ΔFosB proteins, indicators of neuronal activation, in many striatal neurons in LID rats (Ebihara et al., 2011; Bastide et al., 2014; Motojima et al., 2021). In line with previous reports, our result showed that striatal c-Fos^+^ neuron counts strongly correlated with the total AIMs scores. The cell types of the activated c-Fos^+^ neurons are not fully clarified. Various observations suggested that c-Fos and ΔFosB proteins are produced by increased striatal D_1_ dopamine receptor/Protein Kinase A/DARPP-32/ERK signaling (Pavón et al., 2006; Santini et al., 2008; Darmopil et al., 2009). Following these reports, we observed that striatal D1-Cre-mCherry^+^ neurons in LID rats preferentially expressed c-Fos right after levodopa injection. This finding is important in seeking specific types of striatal neurons that mediate LID. Consistent with these results, electrophysiological data from LID mice showed that levodopa increased striatal D_1_^+^ neuronal firing rates (Ryan et al., 2018). It should be noted that a minority of c-Fos^+^ striatal neurons were negative for D1-Cre-MCherry in LID rats after the administration of levodopa (Figure 1E). As shown in Supplementary Figure 2, AAV5-hSyn-DIO-mCherry was not successfully expressed in all D_1_^+^ neurons. Therefore, some c-Fos^+^ & D_1_ positive neurons showed no mCherry expression in the assay. In addition, it has been reported that ΔFosB accumulation occurred in a few D_1_-negative medium spiny neurons and interneurons in the dorsolateral striatum of LID animals (Pavón et al., 2006; Engeln et al., 2016). This supposes that a non-negligible minority of D_1_-negative neurons are activated too upon levodopa treatment. Moreover, it is noteworthy that the percentage of c-Fos positive neurons in D1-Dre-mCherry^+^ neurons was not high after levodopa injection in the present study. We attribute the result to the dose of levodopa in the study. In contrast to previous studies (Pavón et al., 2006; Darmopil et al., 2009), we used a low dose of levodopa (6 mg/kg, s.c) in our experiment to reduce the side effects of levodopa in rats.

All aforementioned findings demonstrate that striatal D_1_^+^ neurons are activated during LID, but they do not prove whether these neurons directly mediate dyskinesia. An increase in the single-unit activity of striatal D_1_^+^ neurons occurred during the dyskinetic phase in LID mice (Ryan et al., 2018). However, the data did not show a direct connection between single-unit activity of striatal D_1_^+^ neurons and each dyskinetic movement. It has been reported that body movement can be predicted based on the population activity of motor cortical neurons (Georgopoulos et al., 1986, 1988). Therefore, we hypothesized that abnormal striatal D_1_^+^ neuronal population dynamics might mediate dyskinetic behavior in LID. Fiber photometry enables quantification of the relationship between neuronal population activity and real-time behavior (Gunaydin et al., 2014; Kim et al., 2016). In the present study, we used GCaMP_6_-based fiber photometry to explore the real-time role of striatal D_1_^+^ neuronal population dynamics in the dyskinetic behavior of LID rats. The results showed that the average power of the striatal D_1_^+^ neuronal GCaMP fluorescence signal in LID rats was significantly higher than that in controls. In addition, we observed that the population activity of striatal D_1_^+^ neurons peaked during each dyskinetic movement in LID rats, suggesting that the aberrant population activity of striatal D_1_^+^ neurons was synchronized with the dyskinetic movement of LID rats. However, to determine a causal link between striatal D_1_^+^ neuronal activity and levodopa-related dyskinesia, we must be able to manipulate the neuronal activity of striatal D_1_^+^ neurons in LID rats and observe the behavioral changes after the manipulation.

Using optogenetic inhibition of striatal D_1_^+^ neurons in D_1_-Cre LID rats, we tested whether deactivation of striatal D_1_^+^ neurons could directly affect levodopa-induced dyskinetic behaviors. In D_1_-Cre LID rats injected with eNpHR3.0, yellow light effectively inhibited most of the dyskinetic symptoms including locomotive, axial, and limb dyskinesias following levodopa administration. These findings consistent with those from a previous report showing that optical stimulation of striatal D_1_-expressing MSNs induced dyskinesia-like behavior in a mouse model of PD (Perez et al., 2017). These results suggest that aberrant striatal D_1_^+^ neuronal population overactivity encodes dyskinetic information and is necessary for the expression of dyskinetic symptoms. The pathogenesis of this abnormal activity of striatal D_1_^+^ neurons in LID is not clear and is possibly multifaceted. The striatum is embedded in a complex network of inputs, including dopaminergic inputs from the SNc and glutaminergic afferents from the cortex and thalamus (Figure 4B; Alexander and Crutcher, 1990). The abnormal dyskinetic action of levodopa might indicate maladaptive plastic neuronal effects occurring at both presynaptic and postsynaptic levels (Calabresi et al., 2010).

The eNpHR3.0-based optogenetic silencing techniques have expanded the causal understanding regarding the functions of diverse neuronal cell types (Gradinaru et al., 2010). Unfortunately, a major drawback of eNpHR_3.0_ is related to its prominent inactivation over a longer period (>15 s), which renders it unsuitable for applications that require long-lasting silencing (Wiegert et al., 2017; Zhang et al., 2019). In our experiment, eNpHR3.0 based optogenetic inhibition failed to affect the dyskinetic symptoms for a long period (>3 min) even by using discontinuous yellow light illumination. An optimized toolbox for optogenetic deactivation and photo-stimulation protocols is required for further exploration (Mattis et al., 2011; Zhang et al., 2019). For manipulation of the activity of striatal D_1_^+^ neurons over a longer period, chemogenetic tools were introduced into the present study. Unlike eNpHR, which silences neurons *via* strong short-term hyperpolarization, hM4Di-DREADDi induces a modest hyperpolarization of neurons over a longer period (Stachniak et al., 2014; Roth, 2016). We observed that chemogenetic silencing of striatal D_1_^+^ neurons using CNO injection reduced the dyskinetic behavior of hM4Di-expressing LID rats slightly but significantly following levodopa administration over a longer period. These results provide further support to the relevance of abnormal striatal D_1_^+^ neuronal population activity in LID.

## Conclusion

Our data indicated that aberrant activity of the striatal D_1_^+^ neuronal population is causally linked with the real-time dyskinetic symptoms in LID rats, suggesting that these neurons could serve as an ideal target for LID treatment.

## Data Availability Statement

The raw data supporting the conclusions of this article will be made available by the authors, without undue reservation.

## Ethics Statement

The animal study was reviewed and approved by the Ethics Committee for Animal Experimentation of Xi’an Jiaotong University.

## Author Contributions

YW and JL conceived the study. YW and LY designed the experiments. SG, JF, WL, YZ, and QZ performed the experiments. SG, RG, and YW performed statistical analyses. YW and RG wrote the manuscript after a fruitful discussion with JL, SG, and LY. All authors have read and approved the final manuscript.

## Conflict of Interest

The authors declare that the research was conducted in the absence of any commercial or financial relationships that could be construed as a potential conflict of interest.

## Publisher’s Note

All claims expressed in this article are solely those of the authors and do not necessarily represent those of their affiliated organizations, or those of the publisher, the editors and the reviewers. Any product that may be evaluated in this article, or claim that may be made by its manufacturer, is not guaranteed or endorsed by the publisher.

## Supplementary Material

Supplementary Figure 1 Representative histology figures (**A–C**; scale bar = 50 μm) of c-Fos immunoreactivity in the striatal brain section of D_1_-Cre-mCherry LID rats after saline administration. Str, striatum.

Supplementary Figure 2 Representative histology figures (**A–C**; scale bar = 50 μm) and Quantification (**D**; green: D_1_^+^ cell number, red: D_1_-Cre-mCherry^+^ cell number, yellow: percentage of D_1_^+^ neurons positive for D_1_-Cre-mCherry; *n* = 8) of D_1_ immunoreactivity in the striatal brain sections of D_1_-Cre rats after intra-striatal injection of AAV5-DIO-mCherry.

Supplementary Video 1 A replay of simultaneous GCaMP_6m_-based fiber photometry signal (raw data) from striatal D_1_^+^ neurons and real-time behavioral video of a D1-Cre rat with levodopa-induced dyskinesia (LID). D_1_-Cre LID rats were placed individually in the center of the box at 30–80 min after levodopa administration. The animals received an intra-striatal infusion of AAV5-EF1a-DIO-GCaMP6m-hGH-pA at 4 weeks before the test.

Supplementary Video 2 A video showing suppression of dyskinetic movements in a D1-Cre rat with levodopa-induced dyskinesia (LID) following eNpHR3.0-based optogenetic inhibition of striatal D_1_^+^ neurons. D1-Cre LID rats were placed individually in the center of the box at 50–70 min following levodopa administration, and their dyskinetic behavior was tracked for 9 min in optogenetic inhibition tests with 3 min of light inhibition (589 nm, 10.5–s ON/2–s OFF pulse, 0.08 Hz) applied from 3 min after the start. The animals received an intra-striatal infusion of AAV5-EF1a-DIO-eNpHR3.0-eYFP-WPRE-pA at 4 weeks before the test.

## Abbreviations

LID: levodopa-induced dyskinesia
PD: Parkinson’s disease
SNc: substantia nigra pars compacta
MSNs: medium spiny projection neurons
NA: numerical aperture
AIMs: Abnormal Involuntary Movement scale
pMAT: Photometry Modular Analysis Tool
PSD: power spectral density
CNO: Clozapine-N-oxide
ANOVA: analysis of variance
PETHs: peri-event time histograms
DREADDi: G_*i*_-coupled designer receptor exclusively activated by designer drugs
6-OHDA: 6-hydroxydopamine
Mfb: medial forebrain bundle
TH: tyrosine hydroxylase
APO: apomorphine
Cre: cyclization recombinase
AAV: adeno-associated virus
VTA: ventral tegmental area
Sal: saline.
