# Supplementary figures and images for: Striatal D1 Dopamine Neuronal Population Dynamics in a Rat Model of Levodopa-Induced Dyskinesia
Source: Front Aging Neurosci. 2022 Feb 3;14:783893. doi: 10.3389/fnagi.2022.783893 (PMC8850470; doi:10.3389/fnagi.2022.783893)

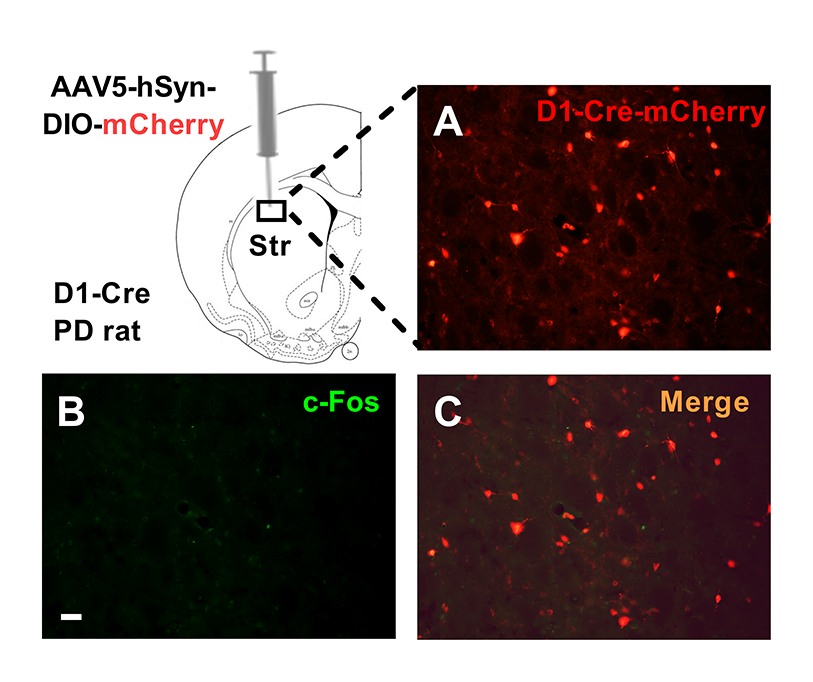

Supplement: Supplementary Figure 1 — Representative histology figures (A–C; scale bar = 50 μm) of c-Fos immunoreactivity in the striatal brain section of D1-Cre-mCherry LID rats after saline administration. Str, striatum. [file Image_1.TIF]

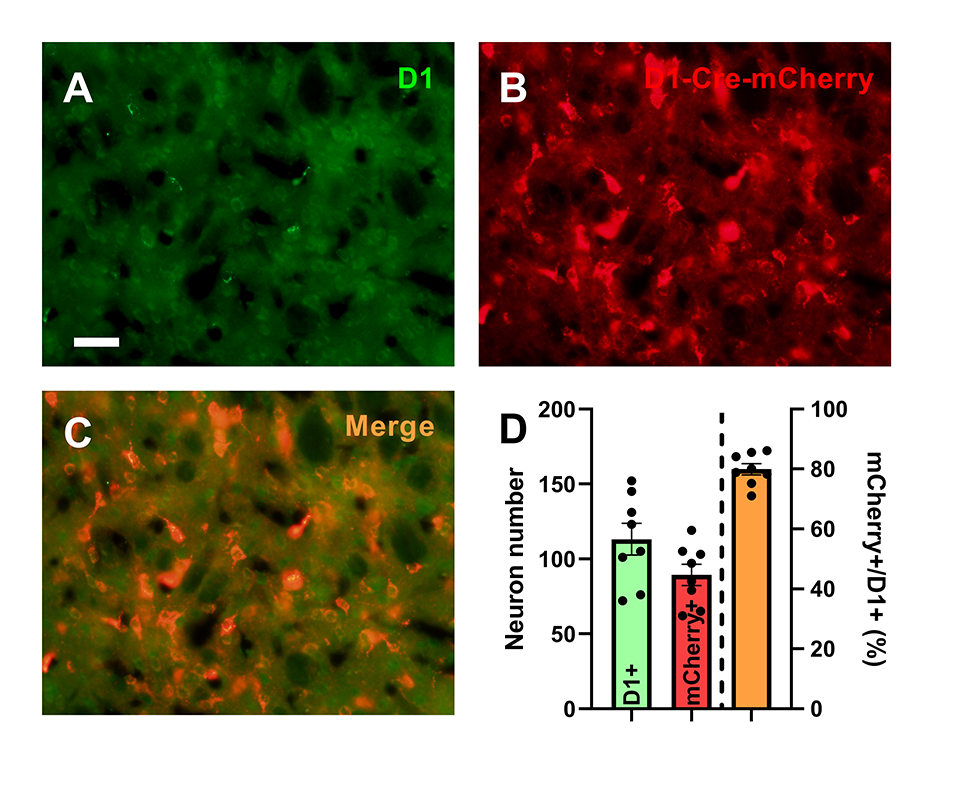

Supplement: Supplementary Figure 2 — Representative histology figures (A–C; scale bar = 50 μm) and Quantification (D; green: D1+ cell number, red: D1-Cre-mCherry+ cell number, yellow: percentage of D1+ neurons positive for D1-Cre-mCherry; n = 8) of D1 immunoreactivity in the striatal brain sections of D1-Cre rats after intra-striatal injection of AAV5-DIO-mCherry. [file Image_2.TIF]
